# Bronchial artery embolization for hemoptysis caused by metastatic hepatocellular carcinoma

**DOI:** 10.1038/s41598-022-10972-9

**Published:** 2022-04-28

**Authors:** Sungwon Kim, Jin Hyoung Kim, Gi-Young Ko, Dong Il Gwon, Ji Hoon Shin, Hyun-Ki Yoon

**Affiliations:** Department of Radiology and Research Institute of Radiology, Asan Medical Center, University of Ulsan College of Medicine, Olympic-Ro 43-Gil, Songpa-Gu, Seoul, 05505 Korea

**Keywords:** Diseases, Oncology, Signs and symptoms

## Abstract

Because of its extremely rare incidence, the safety and efficacy of bronchial artery embolization (BAE) for the treatment of hemoptysis caused by pulmonary metastasis from HCC are not well known. We therefore evaluated the safety and efficacy of BAE in these patients. Data from 18 patients with hepatocellular carcinoma (HCC) and pulmonary metastasis who received BAE for the treatment of hemoptysis between 2003 and 2021 were retrospectively reviewed. Technical and clinical success were achieved in 100% and 94% of patients, respectively. Of the 18 embolization procedures, six were performed using polyvinyl alcohol (PVA) particles only, five were performed using gelfoam only, three were performed using gelfoam plus microcoils, one was performed using PVA plus microcoils, one was performed using embospheres, one was performed using lipiodol plus PVA and gelfoam, and one was performed using hystoacryl with microballoon protection. In eight patients for whom CT just before BAE and at follow-up were available, the mean size of the largest metastatic tumor decreased from 5.1 to 3.7 cm (*P* = 0.035). Hemoptysis recurred in three patients (17%) during follow-up. The median overall and hemoptysis-free survival periods were 149 days and 132 days, respectively. BAE is an effective and safe option for the treatment of hemoptysis in patients with pulmonary metastasis from HCC, with a favorable clinical success rate and a low rate of hemoptysis recurrence. In addition, we also observed BAE to have a positive antitumor effect on pulmonary metastases from HCC, but this requires confirmation in a future study.

## Introduction

With the introduction of regular follow-up examinations for high risk groups, the detection rate of early-stage hepatocellular carcinoma (HCC) has increased, resulting in an increase in the number of patients eligible for curative treatment and increased long-term survival^1^. However, 10–20% of patients receiving curative treatment suffer from extra-hepatic metastases 5–10 years after curative surgery^2^. Furthermore, in those patients whose HCC cannot be cured with radical treatment and who undergo palliative treatment instead, the rate of extra-hepatic metastasis is even higher^3^. Moreover, some patients are diagnosed as having super-induced extra-hepatic metastases, even during their first visit for palliative care^2,4^.

Currently, the lungs are considered the most favored organs for metastatic colonization by HCC, accounting for 51% of all extrahepatic metastasis^5,6^. The results of a large-scale population study in patients diagnosed with HCC lung metastasis showed 1-year overall survival and cancer-specific survival rates of 10% and 12.6%, respectively^7,8^.

Hemoptysis is symptomatic of a variety of diseases. Malignant neoplasms such as primary bronchogenic carcinoma, endobronchial metastatic carcinoma (most commonly from melanoma or breast, colon, or renal cell carcinoma), and bronchial carcinoid can all cause hemoptysis. Because of its rarity, the incidence of hemoptysis in pulmonary metastasis of HCC is not well known, with only a few cases being reported in the literature^9,10^. However, peribronchial pulmonary metastasis from HCC can cause life-threatening hemoptysis that is related to poor prognosis when treated conservatively, with a mortality rate exceeding 50%^11^.

Bronchial artery embolization (BAE) is considered a safe and effective option for hemoptysis, regardless of its severity^12–17^. BAE was shown to effectively stop or decrease hemoptysis caused by benign etiology^18–21^. In addition, BAE is also effective for the management of hemoptysis caused by malignancy^22–27^. Because of its extremely rare incidence, the safety and efficacy of BAE for treating hemoptysis caused by metastatic HCC are not well known. To the best of our knowledge, there has been only one case report describing the use of BAE to successfully manage hemoptysis caused by metastatic HCC^10^.

Thus, the aim of this study was to report our experience of BAE in the treatment of hemoptysis caused by metastatic HCC. This study covers the use of the procedure in 18 patients treated in a single large-volume liver cancer center.

## Materials and methods

### Patients

Our institutional review board (institutional review board of Asan Medical Center) approved the study protocol (IRB No. 2021–1558) and waived written informed consent. All methods were performed in accordance with relevant guidelines and regulations.

Between July 2003 and May 2021, 49,050 consecutive patients were diagnosed with HCC at our institution. Among these 49,050 patients, 1346 were identified as having pulmonary metastasis. Among these 1346 patients, 19 were identified as having hemoptysis. Among these 19 patients, one patient developed hemoptysis from nontuberculous mycobacterium infection. Finally, 18 patients (1.3%, 18/1346; all male; mean age, 60.7 ± 9.6 years) who underwent BAE for hemoptysis caused by pulmonary metastasis were included in this retrospective study. In all 18 study patients, the locations of hemoptysis were correlated with HCC lung metastases.

Table 1 shows the patient characteristics. All patients were male and had advanced-stage HCC (Barcelona Clinic Liver Cancer [BCLC] stage C) before undergoing BAE. All patients had previously undergone either curative intent (surgical resection or radiofrequency ablation) or palliative (chemoembolization, sorafenib, or radiotherapy) antitumor treatment. The amount of hemoptysis before initial BAE was considered to be trivial (blood-tinged sputum) in two (11%) patients, moderate (< 300 mL per 24 h) in 10 (56%) patients, and massive (> 300 mL per 24 h) in six (33%) patients.

**Table 1:** Demographics of the study patients.

	All study patients
	(n = 18)
Age, mean ± SD, years	60.7 ± 9.6
**Etiology (%)**	
HBV	13 (72)
HCV	1 (6)
Alcoholic	3 (16)
Unknown	1 (6)
**Child–Pugh class**	
A	10 (56)
B	8 (44)
**Extrahepatic metastasis other than lung**	
Bone	7 (39)
Brain	6 (33)
Adrenal	3 (16)
Peritoneal seeding	2 (11)
Diaphragmatic invasion	1 (6)
PV invasion	6 (33)
HV, IVC, or RA invasion	5 (28)
**Pulmonary metastasis**	
Multiple	17 (94)
Maximal diameter, mean ± SD, cm	4.2 ± 1.8
Tumor with post-obstructive pneumonia or atelectasis	8 (44)

Numbers in parentheses are percentages.

*SD* standard deviation, *HBV* hepatitis B virus, *HCV* hepatitis C virus, *PV* portal vein, *HV* hepatic vein, *IVC* inferior vena cava, *RA* right atrium.

To locate the source of hemoptysis and identify the underlying etiology, chest radiography and chest CT were carried out on all patients except for one who only underwent serial chest radiographs. Four patients received bronchoscopy prior to BAE: three patients received diagnostic bronchoscopy only, whereas the other patient underwent saline lavage and epinephrine treatment in addition to localization of the bleeding site.

Prior to BAE, patients received supportive measures, such as monitoring of cardiorespiratory parameters, correction of hypoxia, stabilization of blood pressure, and transfusion with blood products when necessary. Some patients with massive hemoptysis were managed in an intensive care unit before and after the BAE procedure. But there was no patient who underwent endotracheal intubation.

Indications for embolization included moderate to massive hemoptysis, or small volume hemoptysis refractory to conservative management and/or flexible bronchoscopy. Decision to pursue BAE was made by the primary clinical service (pulmonology or thoracic surgery) in a coordinated interdisciplinary fashion of interventional radiology teams.

### BAE procedure

Femoral artery access was used to perform BAE in all cases. For visualization of the bronchial artery and identification of other nonbronchial systemic arteries, a diagnostic angiography of the aortic arch and descending thoracic aorta was obtained with use of a 5-F multi-side-hole catheter (Pigtail; Cook). Shaped 5-F angiographic catheters (Cobra, Simmons, Headhunter; Cook) was used to obtain selective bronchial angiographies.

A 1.9–2.4-F microcatheter (Progreat, Progreat Lambda, Terumo; ASAHI Tellus, Asahi Intecc; Renegade, Boston Scientific) was coaxially introduced through the 5-F angiographic catheter into the bronchial artery as distally as possible. The choice of embolic material was made at the discretion of the physician’s personal preferences who performed BAE. The embolic materials used for BAE were 355–500 µm polyvinyl alcohol particles (PVA) (Contour, Boston Scientific; Bearing, Merit Medical), 100–300 µm microspheres (Embosphere, Merit Medical), 1 mm-diameter gelfoam slurry (Spongostan, Ferrosan Medical Devices), iodized oil (Lipiodol, Guerbet), NBCA (n-Butyl cyanoacrylate; Histoacryl; BRAUN), and microcoils (Tornado, extended length: 14 cm, diameter: 3 mm, Cook).

### Definitions and data analysis

Outcomes were defined according to the Society of Interventional Radiology Standards of Practice Committee^28^. Technical success was defined as the ability to selectively catheterize and embolize the abnormal bronchial or nonbronchial systemic arteries. Clinical success was defined as complete (cessation of hemoptysis) or partial success (a marked decrease in hemoptysis with a positive effect on the clinical course of the patient) within 48 h. Clinical failure was defined as persistent or recurrent hemoptysis during the admission period. In-hospital mortality was defined as death occurring during the same hospital period as the first BAE. The early recurrence was defined as a recurrence occurred within the first month, and late recurrence was defined as a recurrence occurred 1 month after BAE in the management of hemoptysis^29^. Six month mortality was defined as death occurring during the 6 months following the first BAE. The hemoptysis-free survival period was defined as the time elapsed from the initial BAE to the recurrence of hemoptysis or death from any cause. The overall survival period was defined as the interval between the initial BAE and death from any cause. Major complications were defined as sequelae that were permanent or a cause of death, requiring treatment or prolonged hospitalization. Minor complications were complications that did not require treatment and were self-limited.

When the antitumor effect of BAE was evaluated in those patients who had both chest CT just before BAE and follow-up chest CT (at least 1 month after BAE), the Response Evaluation Criteria in Solid Tumors (version 1.1) was used^30^. In addition, the size of the largest metastatic tumor was compared before and after BAE in these patients.

### Statistical analysis

Survival curves for survival and freedom from recurrence of hemoptysis were constructed according to the Kaplan–Meier method. The Wilcoxon signed rank test was used to evaluate the change in size of the largest metastatic lung tumor. All statistical analyses were performed using SPSS for Windows version 21.0 (SPSS, Inc.). Two-sided *p* values < 0.05 were considered to be statistically significant.

## Results

### Technical and clinical outcomes

**Figure 1:**
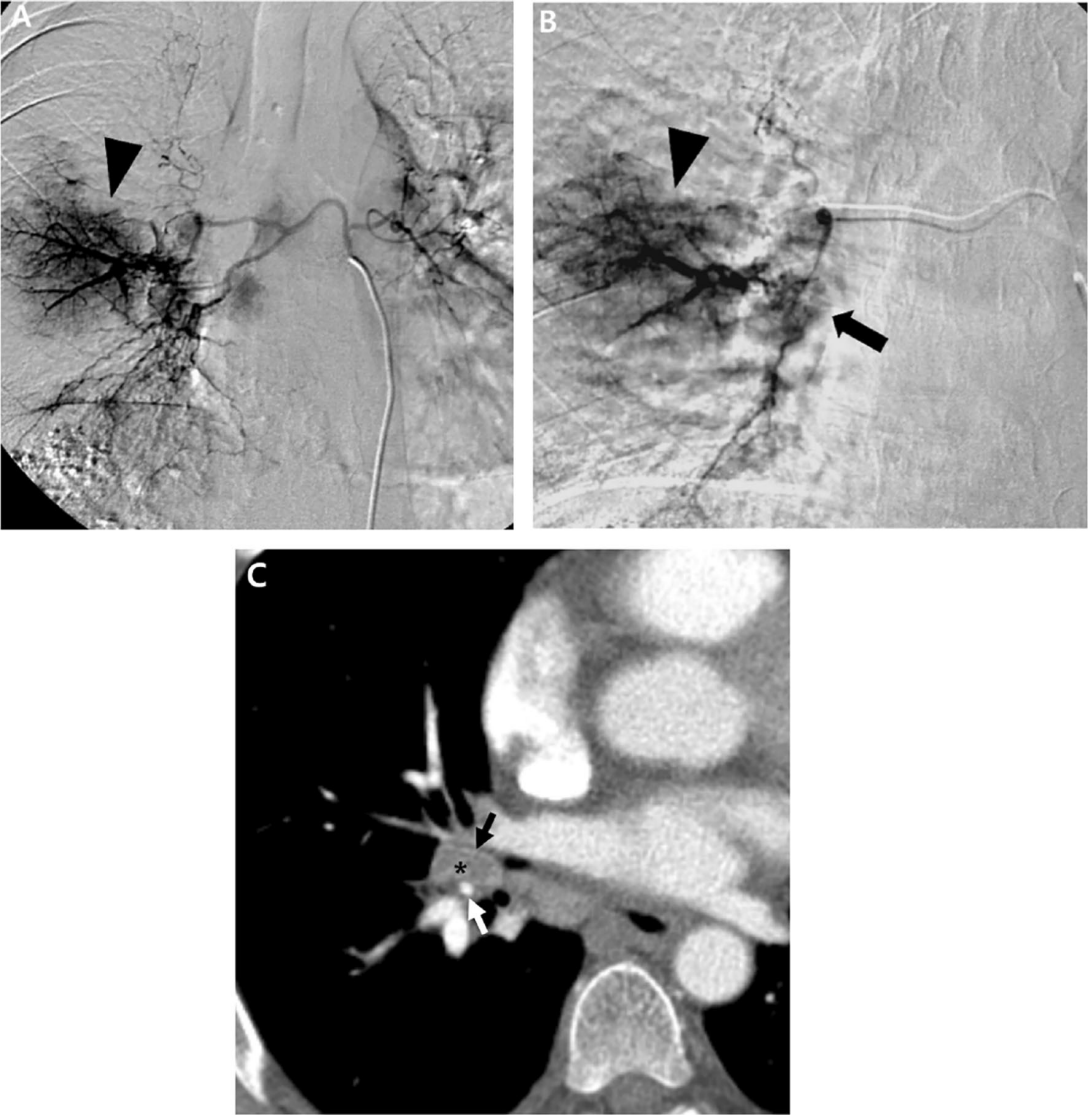
A 56-year-old man with pulmonary metastasis from HCC who presented with a moderate amount of hemoptysis. (**A**) Selective common bronchial trunk arteriography using a 5-F catheter shows multiple mediastinal tumor blushes and an extensive bronchopulmonary shunt (black arrowhead) in the right middle lobe. (**B**) Selective left upper bronchial arteriography using a 2.2-F microcatheter shows a bronchopulmonary shunt (black arrow head) and a metastatic lesion responsible for the tumor blush (black arrow). (**C**) Axial CT image before embolization shows the bronchial artery (black arrow) and pulmonary artery (white arrow) encased by an interlobar metastatic lymph node (black asterisk) between the right middle lobe and right lower lobe bronchus. This finding corresponds with the angiographic finding of a bronchopulmonary shunt.

**Figure 2:**
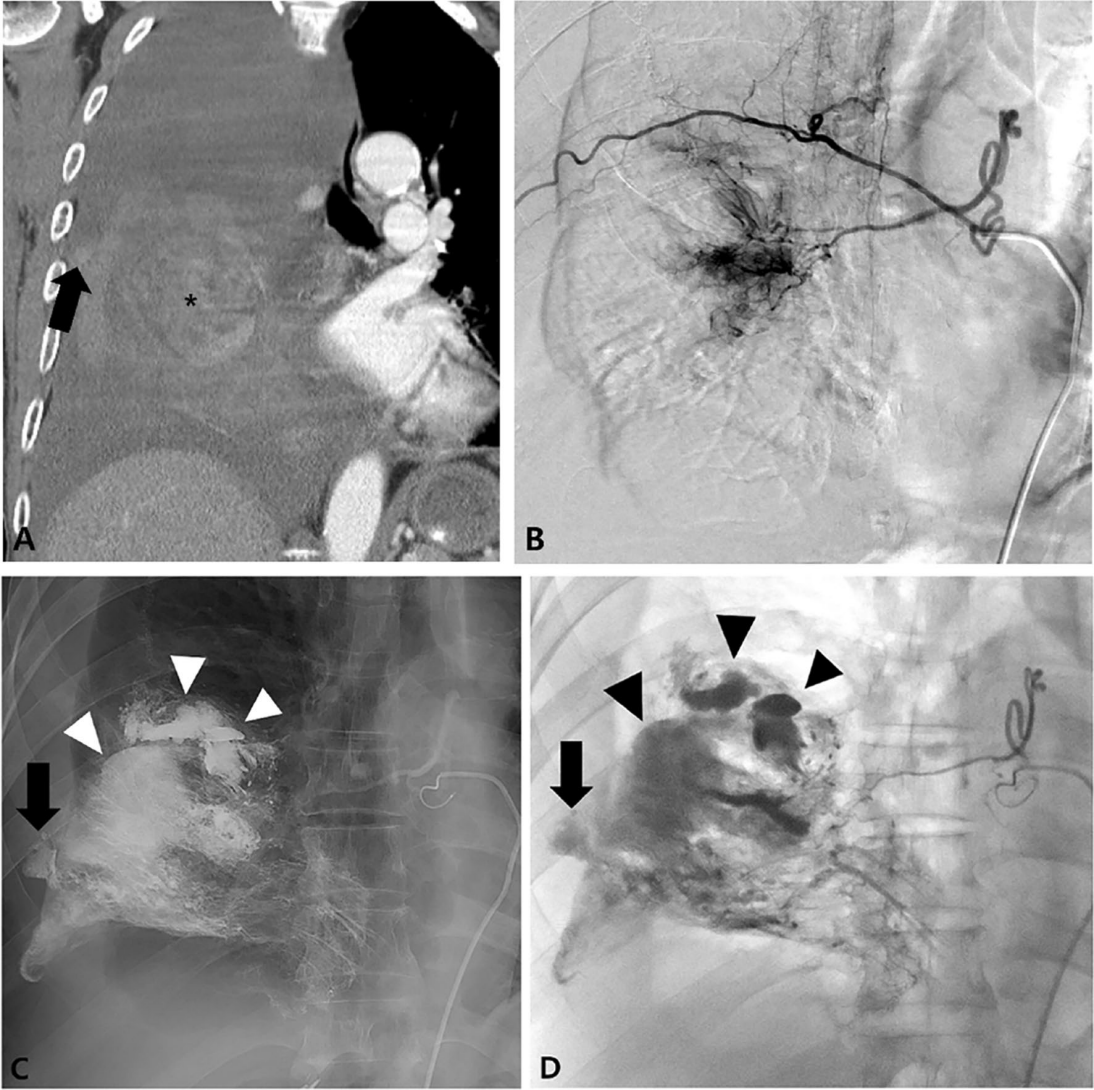
An 80-year-old man with pulmonary metastasis from HCC who presented with a trivial amount of hemoptysis and a large amount of right hemothorax. (**A**) Coronal reconstructed CT image before embolization shows a large enhancing mass (black asterisk) with suspicious contrast media extravasation (black arrow) in the right lower lobe and a large amount of right hemothorax. (**B**)Selective right bronchointercostal trunk arteriography using a 5-F catheter shows a large hypervascular tumor blush fed by the right bronchial artery in the right lower lobe. (**C**–**D**) Embolization of the right bronchial artery was successfully performed using lipiodol, gelfoam, and PVA particles, and this patient showed complete resolution of hemoptysis after bronchial artery embolization. Note the extravasation (black arrow) of lipiodol into the pleural cavity and a vascular lake phenomenon (arrowheads) resulting from intratumoral bleeding.

Details of the BAE procedures and clinical outcomes are shown in Table 2. BAE was technically successful in all 18 patients (100%). Bronchial arteries only were embolized in 16 patients (89%), whereas both bronchial and nonbronchial systemic arteries were embolized in two patients (11%). Of the 18 embolization procedures, six were performed using PVA only, five were performed using gelfoam only, three were performed using gelfoam plus microcoils, one was performed using PVA plus microcoils, one was performed using embospheres, one was performed using lipiodol plus PVA and gelfoam, and one was performed using histoacryl with microballoon protection. Table 3 shows the summary of angiographic findings in all patients and examples are presented in Figs. 1 and 2.

**Table 2:** Details of BAE procedures and clinical outcomes in patients who underwent BAE for the management of hemoptysis caused by pulmonary metastasis from HCC.

Patient no	Hemoptysis volume	Embolized BA or NBA	Embolic material	Clinical response	Second BAE	Survival, days	Cause of death
1	Moderate	RBA, RIMA	PVA, coil	Partial		12	HCC progression
2	Massive	RBA	Gelfoam	Fail	RBA (PVA)	60	HCC progression
3	Moderate	RBA	Gelfoam	Complete		263	HCC progression
4	Moderate	RBA	Gelfoam, coil	Complete		525	HCC progression
5	Moderate	RBA	Gelfoam, coil	Complete		498	HCC progression
6	Trivial	RBA	Lipiodol, PVA, gelfoam	Complete	RBA, LBA (glue, PVA, gelfoam)	394	HCC progression
7	Moderate	RBA, LBA	Gelfoam, coil	Complete		39	HCC progression
8	Moderate	RBA	PVA	Complete		31	HCC progression
9	Massive	LBA	Gelfoam	Complete		193	HCC progression
10	Trivial	RBA, LBA	Gelfoam	Complete		56	HCC progression
11	Massive	RBA	Gelfoam	Complete		481	HCC progression
12	Massive	RBA, LBA	PVA	Complete	LBA (PVA)	149	HCC progression
13	Moderate	RBA, LBA	PVA	Complete		304	Sepsis
14	Trivial	LBA, LICA	PVA	Partial		595	HCC progression
15	Moderate	LBA	Glue with MB protection	Complete		74	HCC progression
16	Moderate	RBA	PVA	Partial		116	HCC progression
17	Moderate	LBA	Embosphere	Complete		68	HCC progression
18	Massive	RBA	PVA	Complete		Alive	

*No.* number, *BA* bronchial artery, *NBA* non-bronchial artery, *RBA* right bronchial artery, *LBA* left bronchial artery, *RIMA* right internal mammary artery, *LICA* left intercostal artery, *PVA* polyvinyl alcohol particles, *MB* micro-balloon catheter, *BAE* bronchial arterial embolization, *HCC* hepatocellular carcinoma.

**Table 3:** Angiographic findings in patients who underwent BAE for the management of hemoptysis caused by pulmonary metastasis from HCC.

Finding	N (%)
Hypervascular tumor blush	18 (100)
Hypertrophic tortuous bronchial or non-bronchial systemic artery	17 (94)
Contrast media extravasation	1 (6)
Bronchopulmonary shunt	2 (11)

Numbers in parentheses are percentages.

Clinical success was achieved in 17 patients (94%): complete clinical success in 14 patients (78%) patients and partial clinical success in three (16%) patients. One patient showed recurrent hemoptysis 2 days after BAE and within the same admission period. A second BAE was performed, with the recanalized right bronchial artery being embolized with PVA. Partial clinical success was achieved after the second BAE, although the patient died of disease progression 2 months later.

**Figure 3:**
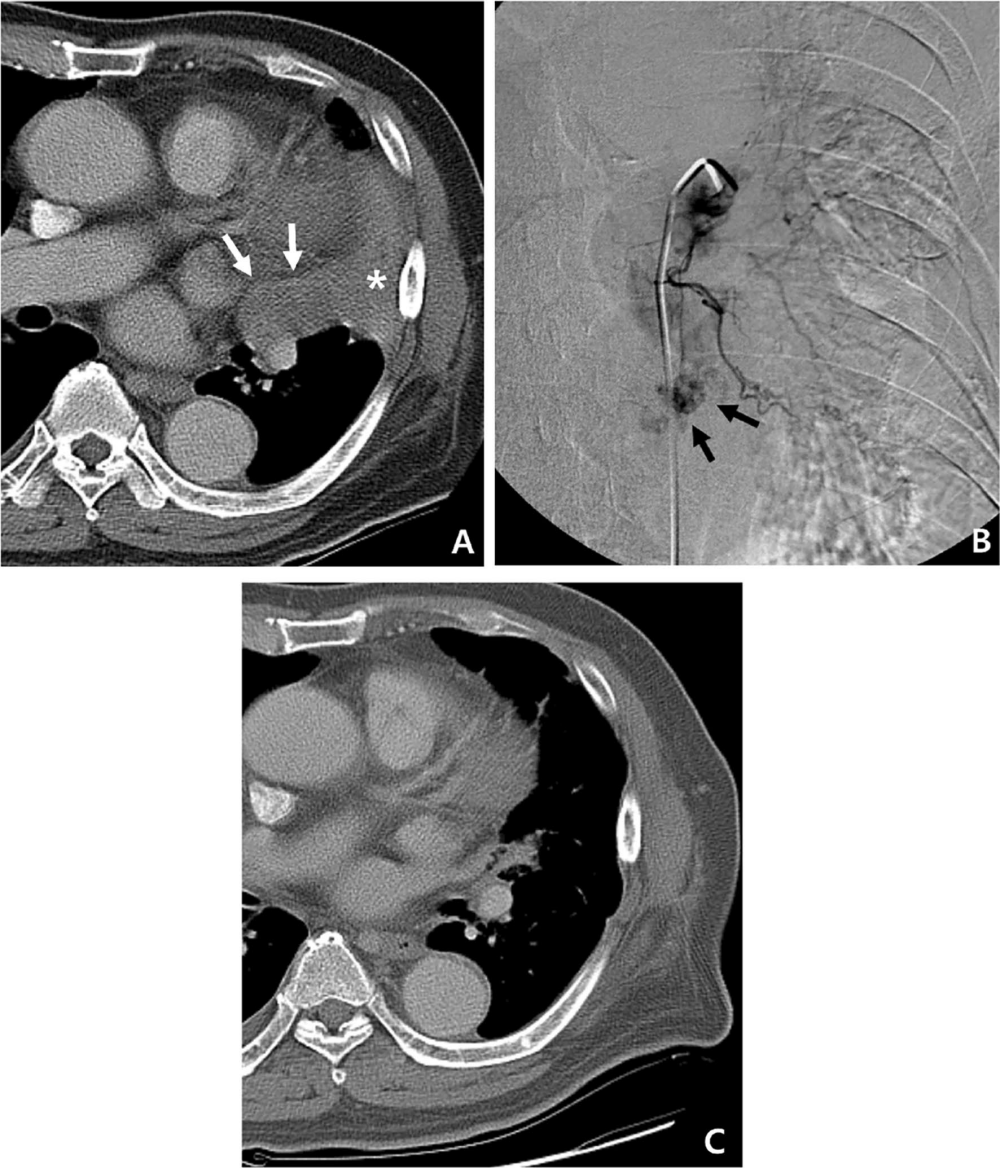
An 81-year-old man with pulmonary metastasis from HCC who presented with a moderate amount of hemoptysis. (**A**) Axial CT image before embolization shows obstructive pneumonitis (asterisk) in the left upper lobe lingular division caused by a metastatic mass (white arrows). (**B**) Selective left bronchial arteriography shows a tortuous hypertrophied left bronchial artery with a hypervascular tumor blush (black arrows) in the proximal portion of the lingular division. Embolization of the left bronchial artery was successfully performed using gelfoam, and this patient showed complete resolution of hemoptysis following bronchial artery embolization. (**C**) Axial CT image 4 months after embolization of the bronchial artery shows the metastatic mass in the lingular division to be markedly decreased in size, as well as an improvement in obstructive pneumonitis.

**Figure 4:**
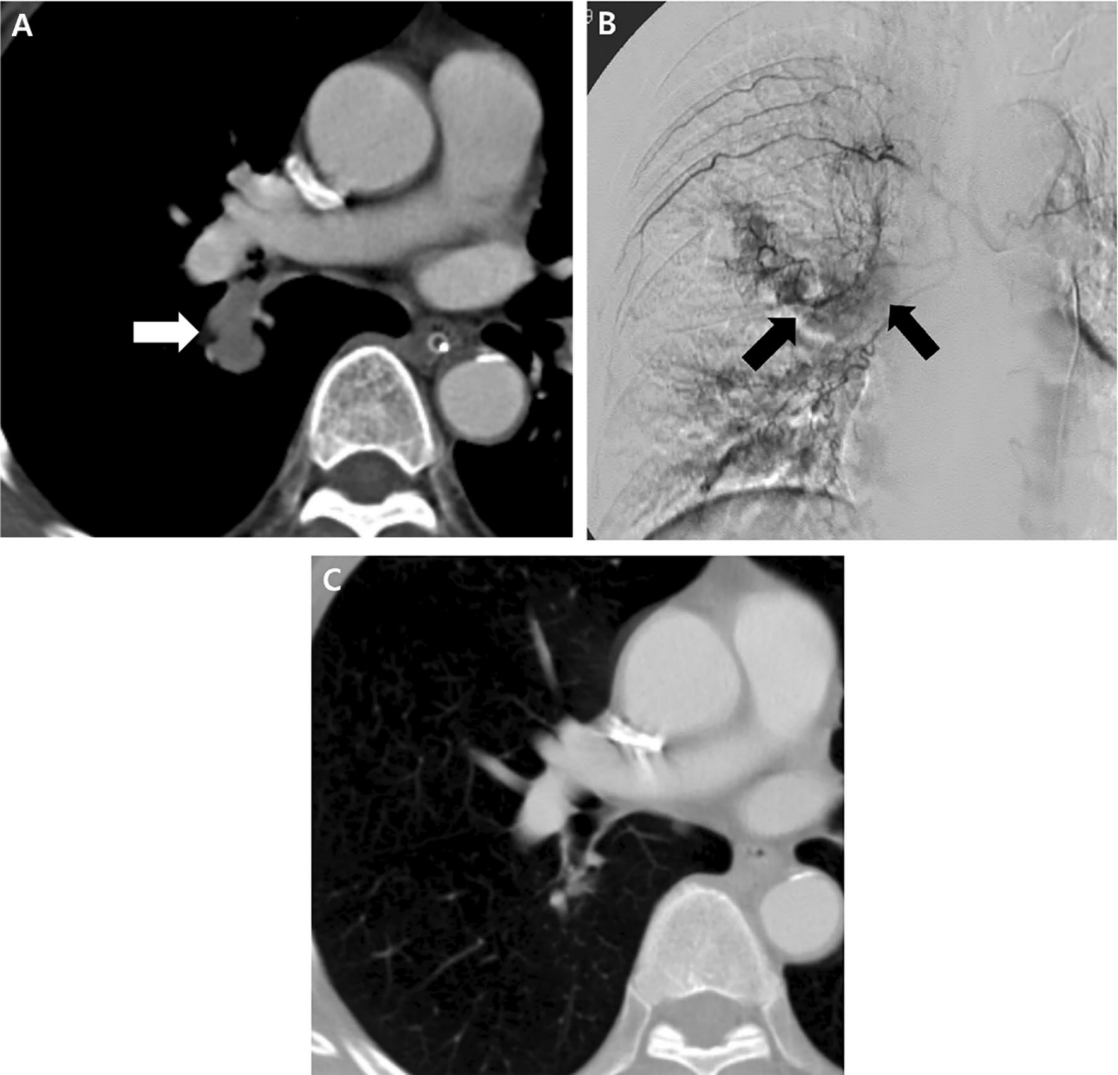
A 72-year-old man with pulmonary metastasis from HCC who presented with a moderate amount of hemoptysis. (**A**) Axial chest CT image before embolization shows an intraluminal soft-tissue mass in the proximal right lower lobe lobar bronchus (white arrow). (**B**) Selective right bronchointercostal trunk arteriography using a 5-F catheter shows multiple hypervascular tumor blushes along the bronchial artery. Note that a hypervascular tumor blush (black arrows) in the proximal right lower lobe lobar bronchus corresponds with endobronchial soft tissue mass in the axial CT image. Embolization of the right bronchial artery was successfully performed using gelfoam and a microcoil, and this patient showed complete resolution of hemoptysis following bronchial artery embolization. (**C**) Axial CT image 4 months after embolization of the bronchial artery shows a marked decrease in the size of the metastatic mass in the right lower lobe lobar bronchus.

**Figure 5:**
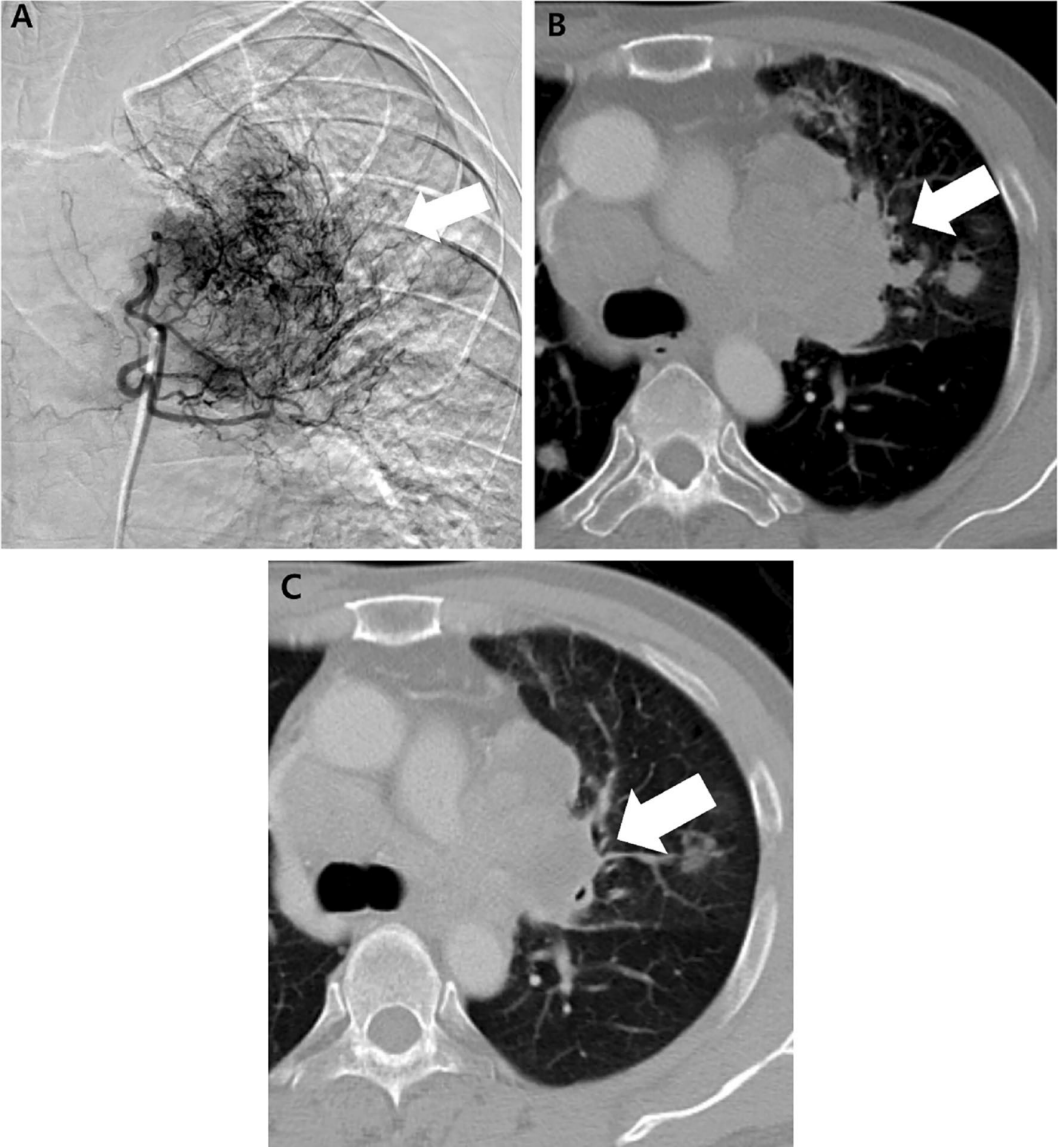
A 63-year-old man with a pulmonary metastasis from HCC who presented with a massive amount of hemoptysis. (**A**) Selective left bronchial arteriography using a 5-F catheter shows a tortuous hypertrophic left bronchial artery and a large hypervascular tumor blush (white arrow). Embolization of the left bronchial artery was successfully performed using gelfoam, and this patient showed complete resolution of hemoptysis after bronchial artery embolization. (**B**) Axial CT image before embolization shows a huge metastatic mass (white arrow) in the left upper lobe. (**C**) Axial CT image 3 months after embolization of the bronchial artery shows a marked decrease in the size of the metastatic mass (white arrow) in the left upper lobe.

**Figure 6:**
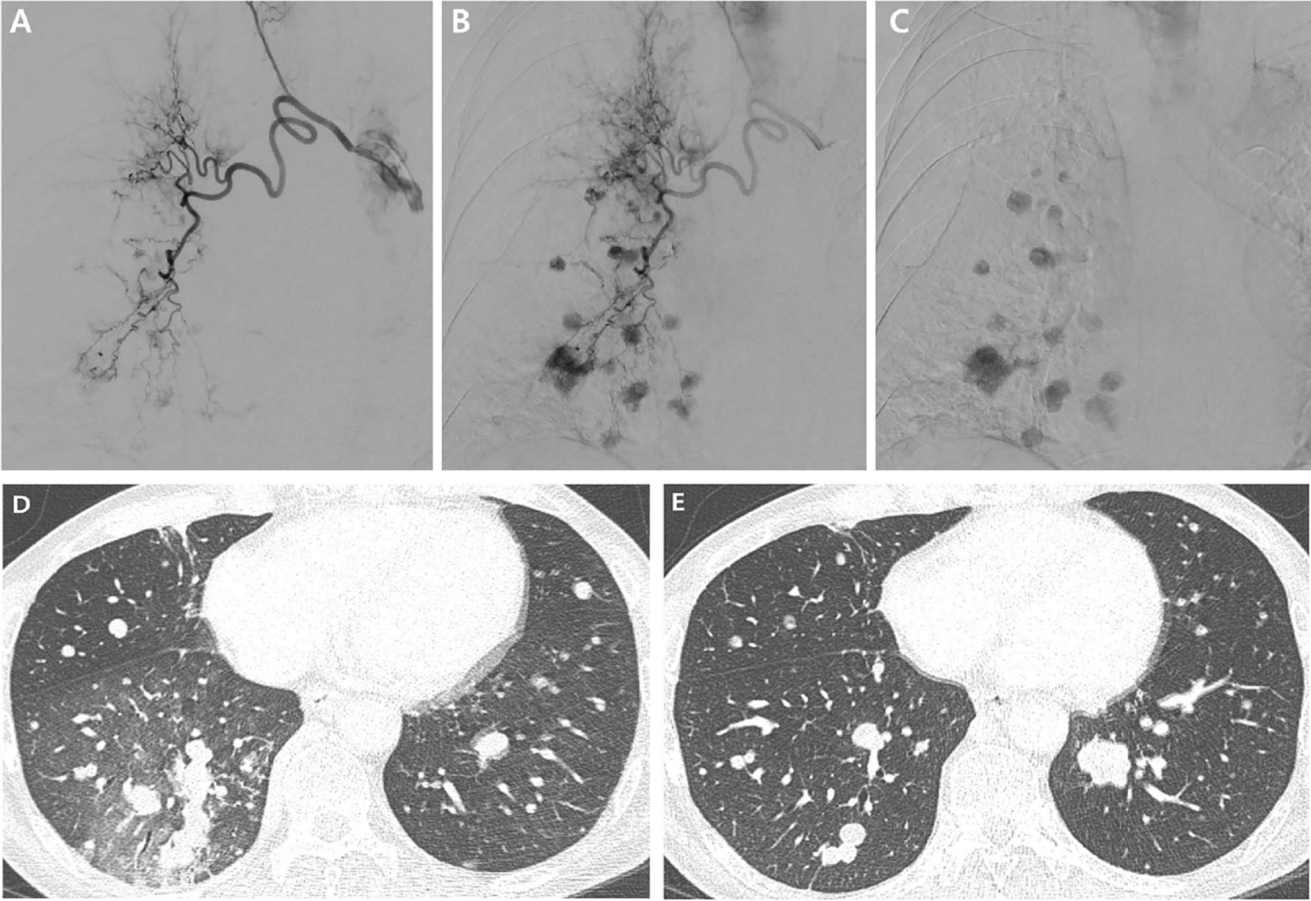
A 57-year-old man with pulmonary metastasis from HCC who presented with a moderate amount of hemoptysis. (**A**–**C**) Selective right bronchial arteriography using a 5-F catheter shows a tortuous hypertrophic right bronchial artery and multiple nodular hypervascular tumor blushes. Embolization of the right bronchial artery was successfully performed using hystoacryl (NBCA: lipiodol = 1:10) and the patient showed complete resolution of hemoptysis after bronchial artery embolization. (**D**) Axial CT image before embolization shows multiple nodular pulmonary metastases in both lungs, particularly prominent in the right lower lobe, with patchy ground glass opacities suggesting aspirated blood. (**E**) Axial CT image 1 month after embolization of the right bronchial artery shows the metastatic nodules in the right lung to be decreased in size and number, but an aggravated pulmonary metastasis that was not in the embolized territory is visible in the left lung.

**Figure 7:**
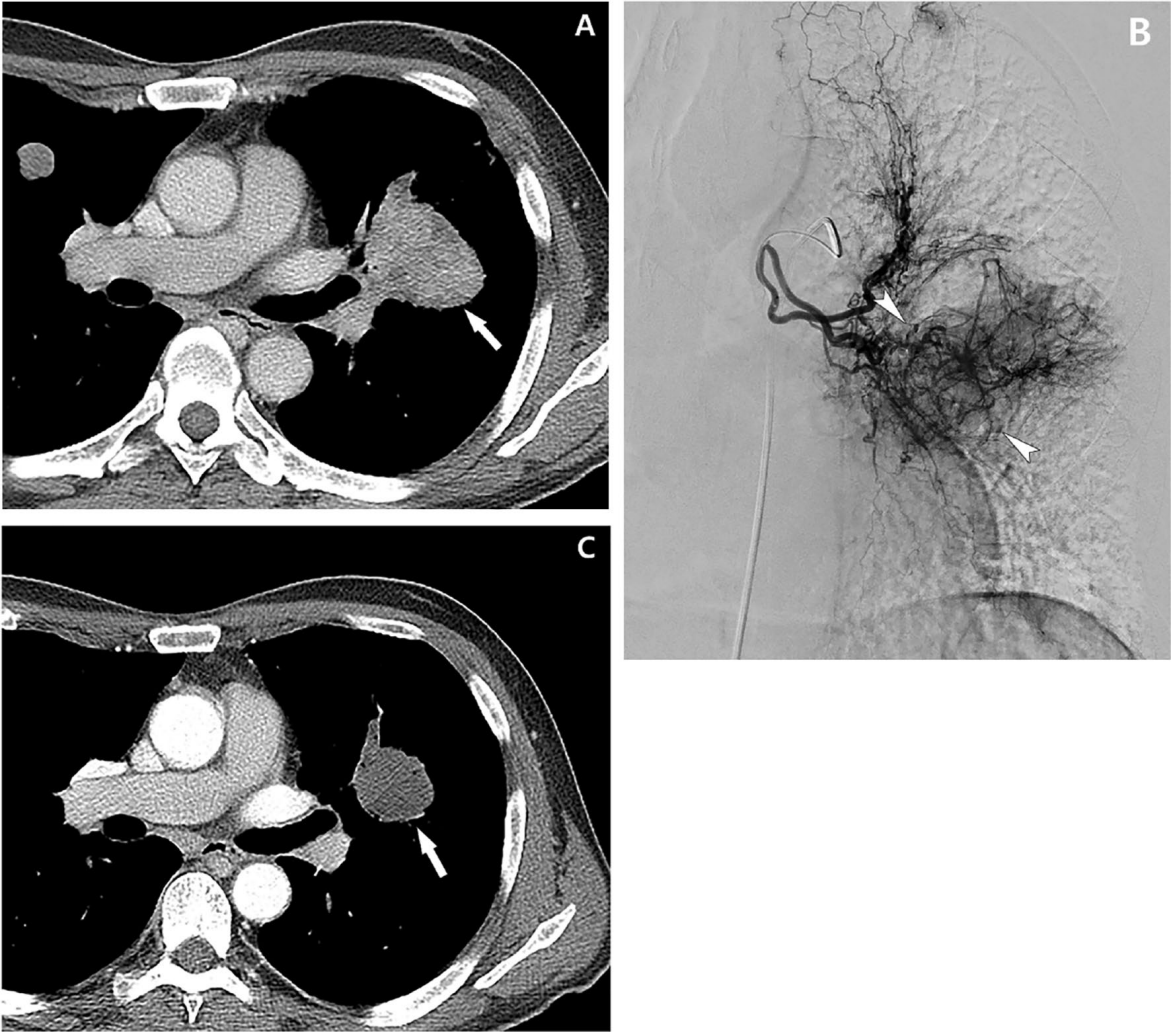
A 61-year-old man with pulmonary metastasis from HCC who presented with a moderate amount of hemoptysis. (**A**) Contrast-enhanced axial CT image before embolization shows a well-enhancing mass (largest metastatic tumor, 5 cm in diameter, white arrow) in the left lower lobe of the lung. (**B**) Selective left bronchial arteriography shows a tortuous hypertrophic left bronchial artery and large hypervascular tumor blush (white arrowheads) corresponding to the largest metastatic tumor on CT. Embolization of the left bronchial artery was successfully performed using PVA, and this patient showed complete resolution of hemoptysis after bronchial artery embolization. (**C**) Contrast-enhanced axial CT image 1 month after embolization of the bronchial artery shows that the largest metastatic mass has decreased in size (white arrow, decreased from 5 to 3 cm in diameter) and seems to be devascularized after bronchial artery embolization.

Of the 18 study patients, evaluation of the antitumor effect of BAE was possible in eight. These eight patients did not receive any antitumor treatment during the period between the pre-BAE CT and the initial follow-up CT. Response of treated metastases one to four months (median, 2 months) after BAE according to the Response Evaluation Criteria in Solid Tumors was partial response in three patients (38%, 3/8), stable disease in four (50%, 4/8), and progressive disease in one patient (12%, 1/8). The mean size of the largest metastatic tumor had decreased from 5.1 cm to 3.7 cm (*P* = 0.035, Wilcoxon signed rank test; Figs. 3, 4, 5, 6 and 7).

Of the 17 patients with initial clinical success, two showed recurrent hemoptysis, 132 and 134 days after initial BAE. In one of these patients who showed a moderate amount of recurrent hemoptysis 134 days after first BAE, follow-up chest CT images and angiography showed progression of pulmonary metastasis and that both bronchial arteries were tortuous and enlarged. The right and left bronchial arteries were successfully embolized using PVA, gelfoam, and hystoacryl, and partial clinical success was achieved. The other patient experienced a massive amount of hemoptysis 132 days after the first BAE. Follow-up chest CT images suggested aggravated endobronchial metastatic tumor, while angiography showed recanalization of the previously embolized left bronchial artery. Complete clinical success was achieved after embolization of the left bronchial artery with PVA.

The overall recurrence rate (one patient with initial clinical failure in the in-hospital period plus two patients with recurrence after initial clinical success) during follow-up was therefore 17%. There was no procedure-related complications.

### Overall survival and hemoptysis-free survival

The in-hospital mortality rate was 11% (2 of 18 patients). In-hospital death was attributed to respiratory failure from progression of lung metastasis in one patient and hepatic failure from progression of primary HCC in the other patient. The 6 month mortality rate was 50% (9 of 18 patients).

**Figure 8:**
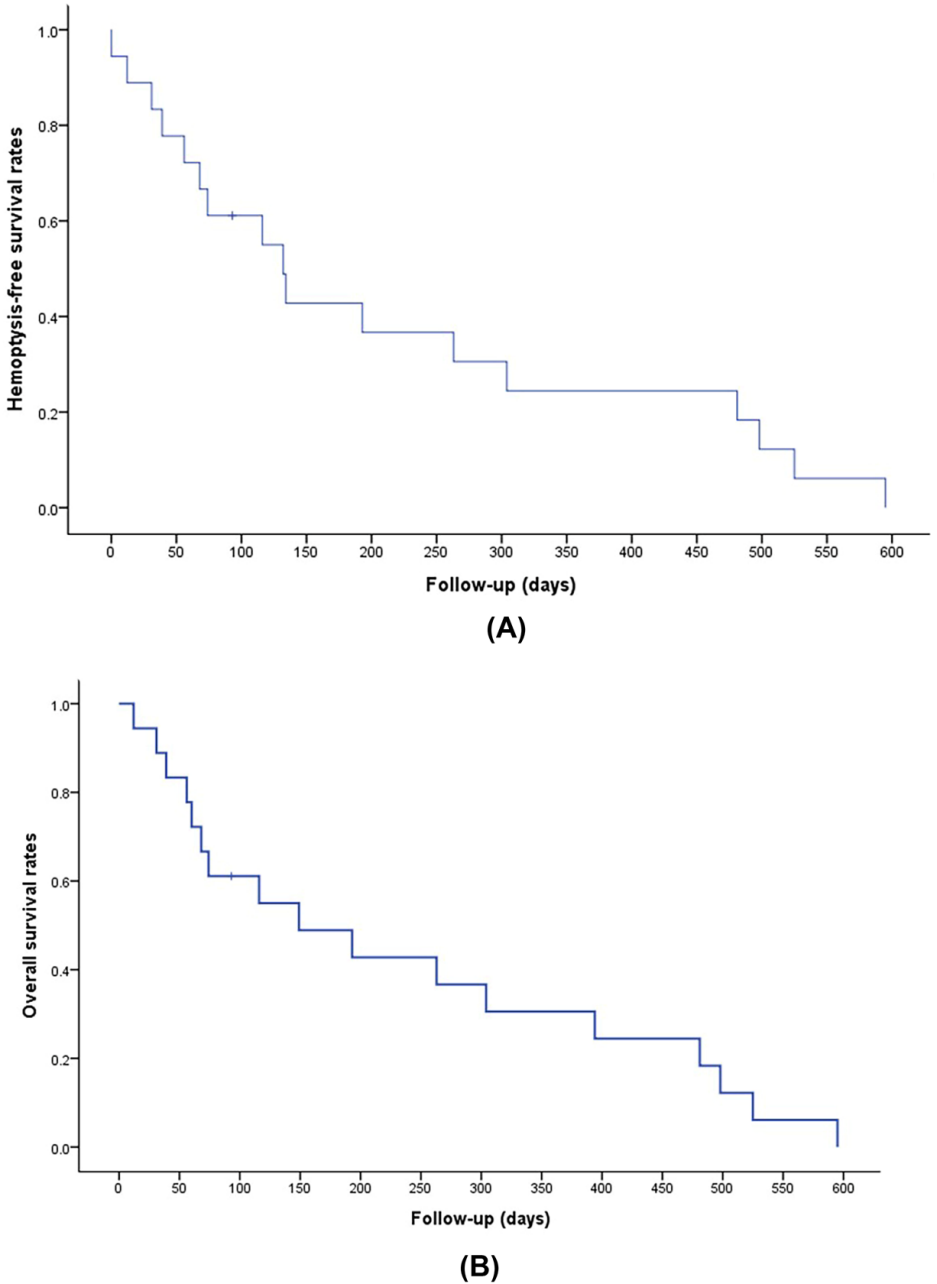
Kaplan–Meier curves showing hemoptysis-free (**A**) and overall survival (**B**) rates.

Of the 18 patients, one was still alive at the time of writing and 17 had died from causes other than hemoptysis. There were 16 deaths attributed to progression of HCC and one death attributed to sepsis. After BAE, the median overall survival was 149 days (95% confidence interval, 0.7–297.3 days) and the median hemoptysis-free survival was 132 days (95% confidence interval, 97.3–166.7 days; Fig. 8).

## Discussion

In the present study, the incidence of hemoptysis requiring BAE was rare, with it occurring in 1.3% of those patients who had metastatic HCC in the lung. We found that BAE was safe and effective at stopping hemoptysis in patients with pulmonary metastasis from HCC. After BAE, technical and clinical success were achieved in 100% and 94% of study patients, respectively. There was no procedure-related complication. In the majority of patients, embolization of the bronchial artery was sufficient to treat HCC-related hemoptysis, and only two patients (11%) required additional embolization of a non-bronchial systemic artery.

During follow-up, three patients (17%; early in one, late in two) in the current study showed recurrent hemoptysis. In the present study, the median overall and hemoptysis-free survival periods after BAE were 149 days and 132 days, respectively. However, considering that all three patients with recurrent hemoptysis were successfully treated with a second BAE, the period with hemoptysis formed a very small portion of the patients’ lives following BAE. There was no hemoptysis-related death in the present study.

An interesting finding in the present study was the positive antitumor effect of BAE in patients with pulmonary metastasis from HCC. The majority of patients showed hypervascular pulmonary tumor staining on angiography, and during the short-term follow-up period after BAE, the mean size of the largest metastatic tumor significantly decreased. In particular, in some patients with endobronchial HCC metastasis, post-obstructive pneumonia improved because of a decrease in the size of the endobronchial tumor. As in the liver, the lung has a dual blood supply, being fed by pulmonary and bronchial arteries. The precise percentages of the blood supplied to metastatic HCC from the bronchial and pulmonary arteries remains unclear, although the majority of the blood supply to pulmonary metastases seems to be from the bronchial artery^31–33^. Chemoembolization or bland embolization is typically indicated for intermediate-stage HCC in the liver^34–37^; however, a recent study showed promising outcomes for chemoembolization in the palliative treatment of pulmonary metastatic tumors from HCC, colorectal cancer, or melanoma^31,33^. As well as chemoembolization, our study may indicate the potential of bland embolization of the bronchial artery in the palliative treatment of metastatic HCC in the lung. However, the role of embolotherapy for pulmonary metastatic HCC still remains poorly defined, and future studies are required to determine the specific patient groups that may benefit from chemoembolization or bland embolization of the bronchial artery in the treatment of pulmonary metastatic HCC.

This study has several limitations. First, it is a retrospective analysis from a single center. Second, the sample size is relatively small and the study period is rather long (> 18 years) because of the low incidence of hemoptysis in patients with pulmonary HCC metastases. Indeed, to the best of our knowledge, no sizable study (except for one case report) has reported the clinical outcomes of BAE in this specific patient group.

In summary, we found that BAE was an effective and safe option for the management of hemoptysis associated with pulmonary metastasis from HCC. In addition, we also observed a positive antitumor effect of BAE on pulmonary HCC metastasis, but this requires confirmation in a future study.
